# Formation and reduction of streak artefacts in electron tomography

**DOI:** 10.1111/j.1365-2818.2009.03357.x

**Published:** 2010-07

**Authors:** M Cao, H-B Zhang, Y Lu, R Nishi, A Takaoka

**Affiliations:** * Key Laboratory for Physical Electronics and Devices of the Ministry of Education, Department of Electronic Science and Technology, Xi'an Jiaotong UniversityXi'an 710049, People's Republic of China; † Research Centre for Ultrahigh Voltage Electron Microscopy, Osaka University7-1 Mihogaoka, Ibaraki, Osaka 567-0047, Japan

**Keywords:** Artefacts, electron tomography, interpolation, transfer function

## Abstract

We have analysed the formation of streak artefacts in the reconstruction based on the filtered back projection algorithm in electron tomography (ET) and accordingly applied an adaptive interpolation technique to artefact reduction. In the adaptive interpolation to recover the missing information, the edge positions in a projection curve were tracked to reduce the interpolation error. A simulation was used to demonstrate the effectiveness of the artefact reduction. Furthermore, image reconstruction of integrated circuit specimens in the ET experiments with the ultra-high voltage electron microscope show that the strong streak artefacts can be reduced effectively by our artefact reduction technique.

## Introduction

As an extremely useful three-dimensional (3D) microscopic imaging tool, electron tomography (ET), has been increasingly developed for obtaining internal structures of nanostructured materials and devices (Kübel *et al.*, 2005; Midgley *et al.*, 2006; Ahrenkiel *et al.*, 2008; Friedrich *et al.*, 2009). At the same time, there are requirements and potentials on improving the reconstruction quality in ET. Here two approaches can be considered—acquiring high-quality two-dimensional (2D) electron microscopic images and improving algorithms for reconstruction. For example, the energy filtered transmission electron microscope, high angle annular dark field imaging and ultra-high voltage electron microscope (ultra-HVEM) have been used to improve image quality (Weyland *et al.*, 2001; Midgley & Weyland, 2003; Takaoka *et al.*, 2008). Recent advances in reconstruction algorithms include the noise reduction, dual-axis iterative algorithm and so on (Frangakis & Hegerl, 2001; Tong & Midgley, 2006; van der Heide *et al.*, 2007).

Nevertheless, streak artefacts are often found in some reconstruction results obtained using the filtered back projection (FBP) algorithm that is widely used in ET. Since artefacts seriously degrade the reconstruction quality, it is crucial to understand why they occur and, more importantly, how they can be prevented or at least suppressed. In X-ray computed tomography, the reconstruction algorithm is a very important factor for artefact formation (Hsieh, 2003) and some artefact reduction methods have been developed (Watzke & Kalender, 2004; Yazdi *et al.*, 2005). However, artefact reduction is still imperative for ET based on the FBP algorithm, especially for recent ET applications in integrated circuit (IC) specimens (Bender *et al.*, 2007; Zhang *et al.*, 2007)

In this work, we analyse the artefact formation from the point of view of signal processing and apply a novel interpolation technique to artefact reduction in ET. Similar with the description of the point-spread function for the tomographic reconstruction (Carazo, 1992; Radermacher, 1992), we first use a transfer function to investigate the information missing and artefact formation. The interpolation methods of recovering missing information are then analysed. A linear interpolation is shown to blur the detailed structure, though it can weaken streak artefacts. Therefore, we propose an adaptive interpolation technique for information recovery and artefact reduction. In this technique, projections of object edges are marked as feature points. The continuous wavelet transform (CWT) technique is used to detect feature points and the shapes near feature points are preserved in the interpolation. A simulation is used to confirm the effectiveness of this technique. More importantly, we show two successful examples of artefact reduction in the ET experiments of IC specimens with the ultra-HVEM. Here we focus our discussion on the 2D image reconstruction because the transverse slices are reconstructed independently in 3D reconstruction.

## Transfer function and artefacts

**Fig. 1:**
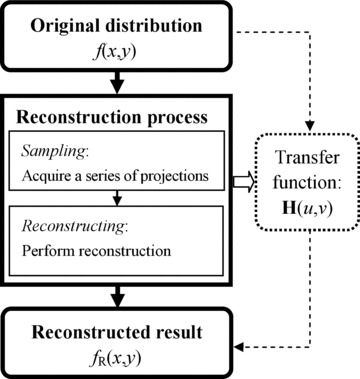
Flowchart of reconstruction process in tomography. The whole process consists of the sampling and reconstructing sub-processes. The original distribution and reconstructed result are the input and output, respectively. The reconstruction process can thus be described by a transfer function.

The essence of ET is reconstructing an object distribution from its projections. As illustrated by the flowchart in Fig. 1, the reconstruction process consists of two sub-processes of sampling and reconstructing and the whole process is equivalent to a linear shift invariant system whose mathematical description is the transfer function **H**(*u*,*v*) (Natterer, 2001). For a perfect system, **H**(*u*,*v*) is unity and the reconstructed result *f*_R_(*x*,*y*) is identical with the original distribution *f*(*x*,*y*). For an imperfect system, however, *f*_R_(*x*,*y*) deviates from *f*(*x*,*y*) because **H**(*u*,*v*) is not unity anymore. Artefacts in the reconstructed result should be related to the imperfection of the transfer function and the artefact formation can be therefore investigated by the properties of the transfer function. The discrete modulation transfer function was given for the reconstructing system based on the FBP algorithm (Glick *et al.*, 1989). Here we do not consider the discrete transfer function since high-resolution projection images in ET can result in negligible difference between the discrete and continuous forms of the transfer function. The continuous form of the transfer function **H**(*u*,*v*) is


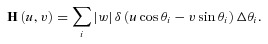
(1)

Here θ*_i_* are projection angles and Δθ*_i_* are their weights.

The transfer function reflects the influences of the sampling and reconstructing sub-processes. From Eq. (1), it can be seen that **H**(*u*,*v*) vanishes unless the point (*u*,*v*) is on the projection lines *u* cos θ_*i*_−*v* sin θ_*i*_= 0. This means that only the information on the projection lines is sampled and other information between projection lines is lost. On the other hand, the delta function in Eq. (1) is modulated by a ramp filter |*w*|, which indicates that the sampled projections are filtered by the ramp filter. The ramp filter works as a differential operator and produces positive and negative pulses at turning points where derivatives are not continuous. These pulses are then projected back onto streaks in the reconstructed image. If the projection interval is fine enough, contributions of positive and negative pulses on closer neighbouring streaks may be counteracted. Otherwise, the unbalanced contributions of pulses should result in considerable streaks in the reconstruction result. Because turning points on projection curves are related to object edges, streak artefacts are tangent to object edges and their directions are the same as those of projections. Moreover, in ET, it is usually difficult to have a very fine projection interval and hence reconstruction qualities are often hampered by streak artefacts.

**Fig. 2:**
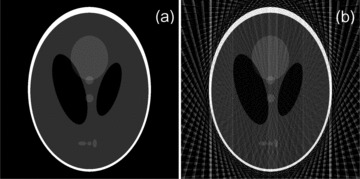
(a) A Shepp–Logan phantom and (b) its reconstructed image from 36 projections uniformly distributed over ±90°. The evident streaks tangent to the edges of objects and along the projection directions can be seen in the reconstruction based on the FBP algorithm.

The analysis of the artefact formation based on the transfer function can be confirmed by a simulation. Here, we use a Shepp–Logan phantom (Shepp & Logan, 1974) shown in Fig. 2(a) as the original image. The reconstruction from 36 projections with a constant interval is shown in Fig. 2(b). As predicted, there are strong streak artefacts tangent to the edges of elliptic objects and the directions of these streaks are the same as those of projections.

## Artefact reduction

**Fig. 3:**
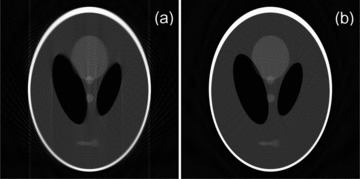
Reconstruction of the Shepp–Logan phantom based on (a) linear interpolation and (b) adaptive interpolation. A total of 180 projections interpolated from the 36 projections were used for reconstruction. Streak artefacts are weakened somewhat but the detailed structures are blurred in (a). In contrast, the artefacts are suppressed effectively in (b) with little influence on the detailed structures.

Recovery of the missing information should reduce the streak artefacts originating from information missing. For this recovery, interpolation is a natural technique. We first tried a linear interpolation. Based on the former 36 projections of the Shepp–Logan phantom, we generated 180 projections with a linear interpolation. These interpolated projections are then used to produce a new reconstructed image. As shown in Fig. 3(a), the streak artefacts become somewhat weaker than those in Fig. 2(b). However, some detailed structures, such as the edges of the ellipses, have been blurred as a result of the simple linear interpolation.

**Fig. 4:**
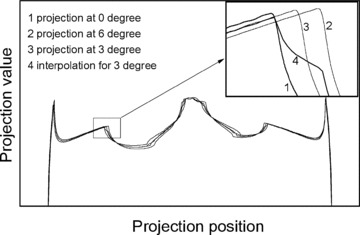
Projections of the Shepp–Logan phantom at 0°, 3°, 6° and linear interpolation for 3° obtained from 0° and 6° projections, noted with number 1–4, respectively. A big error from linear interpolation occurs near the turning point at curve 4, compared with curve 3.

The blurring of detailed structures in the reconstructed image is due to interpolation errors. If a structure is projected onto different positions at different projection angles, a direct interpolation will mix the information of different positions and thus lead to a big interpolation error. The interpolation error finally results in incorrect information in the reconstructed image. We now illustrate this kind of interpolation error with an example. Fig. 4 gives the projection curves of the former Shepp–Logan phantom at 0°, 3° and 6°. The linear interpolation curve for 3° obtained from 0° and 6° projections is also compared in the same figure. The inserted figure gives a detailed scene near a turning point, which is the projection of the left edge of the left dark ellipse in the phantom. On the curves 1 and 2 respectively corresponding to 0° and 6° projections, we can see that the turning points lie on different positions. Hence, the direct interpolation of these two curves effaces the sharp shape and the interpolated curve 4 for 3° deviates from the correct curve 3 near the turning point. This deviation consequently blurs the edge in the reconstruction result.

We now present an adaptive interpolation technique not only for reducing streak artefacts but also for suppressing the blurring of detailed structures. Streak artefacts can be considered to originate from pulses near turning points and the counteraction between neighbouring positive and negative contributions can weaken streak artefacts. Thus, sharp shapes near turning points should be preserved on interpolated curves to reduce streak artefacts. From the inserted figure of Fig. 4, we see that the turning point shifts when the projection angle changes from 0° to 6°. This motion of the turning point should be represented in the interpolated projection sequence. In our adaptive interpolation technique, turning points are first marked as feature points. The features are picked out in pairs on two adjacent projections. For two adjacent projections at projection angles θ_1_ and θ_2_, we assume that there is a pair of feature points *x^k^*_1_ and *x^k^*_2_. To reflect the motion of the feature points, we determine the new corresponding feature point *x^k^* at the projection angle θ by


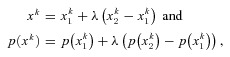
(2)

where 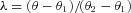 and *p*(*x*) is the projection value at point *x*. After fixing the feature points, the interpolation is then skewed to preserve these features. For an arbitrary point *x* between two adjacent feature points *x^k^* and *x*^*k*+1^ at the projection angle θ, its corresponding points *x*_1_ and *x*_2_ at projection angles θ_1_ and θ_2_ are


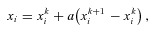
(3)

where *i*= 1 or 2 and 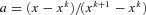. Finally, the projection value *p*(*x*) is given by a linear combination of *p*(*x*_1_) and *p*(*x*_2_)


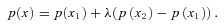
(4)

In the present work, we assume that a feature point moves with a constant speed between two adjacent projections and thus determine the new feature point with a linear interpolation. A further improvement could be calculating new feature points using a suitable interpolation method that describes motions of feature points more precisely.

**Fig. 5:**
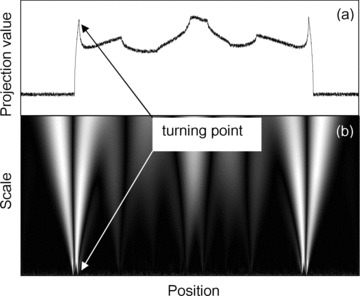
(a) Zero degree projection with noise for the Shepp–Logan phantom and (b) its CWT. Here, the rears of high response area in the small-scale range can give the fine positions of turning points robustly with respect to noise.

Detecting feature points is an important step in this adaptive interpolation technique for reducing artefacts. A general method is to calculate the convolution integral between the projection curve and a certain detection operator. Note that peaks near different turning points may have various scales. The detection operator should therefore be adaptable to different scales. Here the CWT is introduced for feature detection since it possesses the ability to construct a position-scale representation of a signal and can therefore localize both position and scale accurately. The 0° projection curve of the Shepp–Logan phantom and the abstract value of its CWT are shown in Figs 5(a) and (b), respectively. The mother wavelet used here is the Gauss wavelet. To demonstrate the robustness with respect to noise, we added random noises on the projection data. From Fig. 5(b), it can be seen that high responses appear near turning points. In the large-scale range, these responses give rough positions of turning points but suppress the disturbance of noise. The response ridges become finer when they extend to the small-scale range. The corresponding rears in the small-scale range can thus give fine positions of turning points.

Using the presented adaptive interpolation technique, we have again performed an image reconstruction for the Shepp–Logan phantom. A total of 180 projections were generated from the former 36 projections and the reconstructed image based on the 180 projections is shown in Fig. 3(b). Compared with Figs 2(b) and 3(a), the streak artefacts are suppressed effectively. It can be noted that the three small ellipses at the bottom are still somehow blurry. One reason for this blurring is that projections of this kind of small objects with low contrasts are merged in projections of other objects and become undistinguishable. Another reason is that the ellipses are far away from the tilt axis and the assumption of constant speed is unsuitable for their motions.

**Fig. 6:**
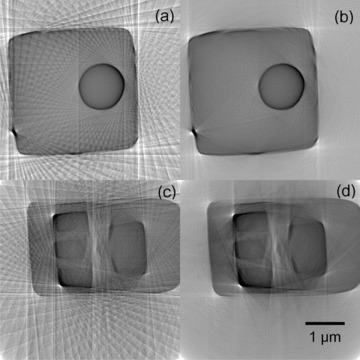
Reconstruction of a via in an IC specimen based on (a) FBP algorithm and (b) adaptive interpolation, and buried copper lines in the other IC specimen based on (c) FBP algorithm and (d) adaptive interpolation. In (b) and (d), 180 projections interpolated from 36 projections uniformly distributed over ±90° were used for image reconstruction so that the strong artefacts have been reduced considerably.

The efficiency of the artefact reduction technique can be also validated by ET experiments of IC specimens. Rod-shaped specimens were prepared using a focused ion beam and then a tilt series of transmission electron microscopic images were collected using the ultra-HEVM. The specimen were titled at an interval of 1° over the range from −90° to 90° and 180 images were recorded with a high performance cooled charge coupled device camera. To demonstrate properties of the artefacts and the effectiveness of the reduction, we selected projection subsets with a projection interval of 5° for the reconstructions. Two cross-sections of different IC specimens were demonstrated here. The cross-sections are perpendicular to the tilt axis. One is a cross-section of a via in the IC specimen (Cao *et al.*, 2009). The directly reconstructed image using the FBP algorithm is shown in Fig. 6(a). Strong streak artefacts due to information missing can be found. The reconstructed image with artefact reduction is shown in Fig. 6(b). Here we calculated 180 projections from the former 36 projections using the adaptive interpolation and then performed the image reconstruction based on the interpolated projections. It can be seen that the artefacts are suppressed effectively. The cross-section of another specimen which includes some buried copper lines (Zhang *et al.*, 2007) is shown in Figs 6(c) and (d). It is evident that most of the strong streak artefacts in Fig. 6(c) become much weaker in Fig. 6(d). Note that the remaining artefacts in Fig. 6(d) are stronger than those in Fig. 6(b). This is because that the first structure contains only high contrast edges that are easy to be tracked. The low contrast edges of small objects in the second structure are difficult to be tracked. Also it is difficult to have a high-quality 2D microscopic image for small objects with low contrasts. Nevertheless, our technique can suppress most of the strong streak artefacts with little damage on the information near the object edges.

The presented technique for artefact reduction is particularly useful for the ET reconstructions of the non-biological specimens such as IC specimens or other nanostructured device specimens that often contain many high contrast edges. Intense pulses are created around these edges by the ramp filter and this may consequently lead to evident streak artefacts in the reconstructed image. On the other hand, the high contrast edges are easier to detect and hence the streak artefacts can be reduced more effectively. In addition, the structures of non-biological specimens are often determined by the edge of objects. The special treatments on the edges of objects will help to provide clearer views of structures.

The extra computing work introduced by the given artefact reduction technique can be reduced by interpolating the back projections. In the FBP algorithm, a time-consuming work is calculating back projections from projections. Although our method needs more projections, the required back projections can be directly calculated by interpolation. The positions of feature points and interpolation technique for back projections are the same as those for projections. Hence, calculating back projections from the interpolated projections is not necessary anymore.

Another kind of important issue in ET is the missing wedge problem due to the limited tilt angle range. To recover the information in the missing wedge, an extrapolation has to be used. However, the extrapolation is usually not as stable as the interpolation. A possible method to overcome the missing wedge problem would be acquiring more projections, such as a dual axis tomography (Arslan *et al.*, 2006). Nevertheless, ET of the rod-shaped specimens in this work does not suffer from such problem because of the complete tilt range of ±90°.

An ideal approach to reverse the effect of the transfer function on the reconstruction quality is deconvolution of the determined transfer function, such as the method of constrained maximum entropy tomography (Skoglund *et al.*, 1996). However, a time-consuming iterative refinement is often required for deconvolution. The presented artefact reduction technique in our work keeps the advantage of the fast speed of the FBP algorithm by recovering the missing information.

## Conclusions

Based on the analysis of the artefact formation, we addressed the reduction of the streak artefacts that were caused by the information missing due to an imperfect transfer function. An adaptive interpolation technique of tracking the feature points was proposed and applied to recover the missing information and to reduce streak artefacts. Both the simulation and the results of ET experiments of IC specimens with different structures show that this technique is effective for reducing streak artefacts but not at the expense of information of detailed structures and therefore improving the reconstruction quality in ET.
